# Protective Effect of *Lactobacillus diolivorans* 1Z, Isolated From Brazilian Kefir, Against *Salmonella enterica* Serovar Typhimurium in Experimental Murine Models

**DOI:** 10.3389/fmicb.2018.02856

**Published:** 2018-12-04

**Authors:** Mario Abatemarco Júnior, Sávio Henrique Cicco Sandes, Mayra Fernanda Ricci, Rosa Maria Esteves Arantes, Álvaro Cantini Nunes, Jacques Robert Nicoli, Elisabeth Neumann

**Affiliations:** ^1^Departamento de Microbiologia, Instituto de Ciências Biológicas, Universidade Federal de Minas Gerais, Belo Horizonte, Brazil; ^2^Departamento de Biologia Geral, Instituto de Ciências Biológicas, Universidade Federal de Minas Gerais, Belo Horizonte, Brazil; ^3^Departamento de Patologia Geral, Instituto de Ciências Biológicas, Universidade Federal de Minas Gerais, Belo Horizonte, Brazil

**Keywords:** *Lactobacillus diolivorans*, kefir, probiotic, gnotobiotic mice, *Salmonella* Typhimurium

## Abstract

Kefir is a beverage obtained by fermentation of milk or sugar solution by lactic acid bacteria and yeasts, and several health benefits have been attributed to its ingestion, part of them being attributed to *Lactobacillus* species. The objective of the present study was to evaluate, *in vivo*, the probiotic potential of *Lactobacillus diolivorans* 1Z, isolated from Brazilian kefir grains. Initially, conventional mice were orally treated daily or not during 10 days with a suspension of *L. diolivorans* 1Z, and then orally challenged with *Salmonella enterica* serovar Typhimurium. Treatment with *L. diolivorans* 1Z resulted in higher survival (70%) of animals after the challenge with the pathogen than for not treated mice (0%). When germ-free mice were monoassociated (GN-PS group) or not (GN-CS group) with *L. diolivorans* 1Z and challenged after 7 days with *S.* Typhimurium, *Salmonella* fecal counts were significantly lower (*P* < 0.05) for the GN-PS group when compared to the GN-CS group. Histopathological analysis revealed less damage to the ileum mucosa, as demonstrated by smallest perimeter of major lesions for mice of the GN-PS group in comparison to the group GN-CS (*P* < 0.05). These findings were accompanied by a lower expression of IFN-γ and TNF-α in the intestinal tissue of GN-PS mice. Additionally, translocation of *S.* Typhimurium to liver was significantly lower in GN-PS than in GN-CS mice (*P* < 0.05), and IgA levels in intestinal content and number of Kupffer cells in liver were higher. No difference was observed for hepatic cellularity between GN-PS and GN-CS groups (*P* > 0.05), but the pattern of inflammatory cells present in the liver was predominantly of polymorphonuclear in GN-CS group and of mononuclear in the GN-PS group, and a higher hepatic expression of IL-10 and TGF-β was observed in GN-PS group. Concluding, *L. diolivorans* 1Z showed to be a potential probiotic strain that protected mice from death after challenge with *S.* Typhimurium, apparently by immunological modulation.

## Introduction

Kefir is an acidic fermented beverage, lightly alcoholic, obtained through the double fermentation (alcoholic and acidic) of milk or sugar solution (Garrote et al., 2010). In the kefir grains, a high microbiological diversity is found which includes yeasts, lactic acid bacteria (*Lactobacillus*, *Lactococcus*, and *Leuconostoc*), acetic acid bacteria, and other microorganisms not yet described (Garrote et al., 2001; Nalbantoglu et al., 2014; Arslan, 2015).

Beneficial effects associated with kefir intake have already been suggested in several studies and there are evidences that many of these effects are attributed to the *Lactobacillus* species (Vinderola et al., 2005; Zamberi et al., 2016; Iraporda et al., 2017; Sharifi et al., 2017). Due to their long history of use in food fermentation and absence of pathogenic factors, *Lactobacillus* are generally recognized as safe for consumption and have been exploited as probiotics, which are live microorganisms that, when administered in adequate amounts, confer a health benefit on the host (FAO/WHO, 2002).

Salmonellosis is an acute infection with a worldwide distribution caused by serovars of *Salmonella enterica* subsp. *enterica*, which are Gram-negative bacteria. Most people with salmonellosis develop diarrhea, fever, vomiting, and abdominal cramps 12–72 h after infection and some patients develop serious complications such as typhoid fever. Oral infection with *S. enterica* subsp. *enterica* serovar Typhimurium in mice provokes a disease similar to that caused by *S. enterica* serovar Typhi in humans, with fever, enteritis (without the acute diarrhea observed in humans), and septicemia which is lethal to the host (Santos, 2014).

In a previous work, we have isolated and identified some *Lactobacillus* strains from Brazilian kefir grains that have been characterized as potential probiotic by *in vitro* tests. In these assays, *Lactobacillus diolivorans* 1Z demonstrated to be resistant to gastric juice and bile salts, to produce antagonistic compounds against several pathogens, and showed an antimicrobial susceptibility pattern typical of *Lactobacillus* genus (Zanirati et al., 2015). These results suggest that *L. diolivorans* 1Z could survive to the passage through the intestinal tract, as expected for a probiotic strain (Ranadheera et al., 2012). This strain was also able to resist to lyophilization process and to produce a heteropolysaccharide that inhibit *S. enterica* serovar Enteritidis adhesion to Caco-2 cells (unpublished data).

The objective of the present study was to evaluate the protective effect of the oral administration of *L. diolivorans* 1Z viable cells on an experimental infection with *S.* Typhimurium in conventional and gnotobiotic (GN) murine models.

## Materials and Methods

### Mice

Twenty-one to twenty-three days old conventional (CV) male mice of the BALB/C lineage were obtained from the Biotério Central, Universidade Federal de Minas Gerais (UFMG, Belo Horizonte, Brazil). Twenty-one to twenty-three days old germ-free (GF) Swiss NIH mice (Taconic Farms, Germantown, United States) were also used. GF animals were kept into flexible plastic isolators (Standard Safety Equipment Co., Palatine, IL, United States). For experiments, CV and GN mice were kept into microisolators (Uno Roestvaststaal B.V., Zavenaar, The Netherlands) maintained in a ventilated animal caging system (Alesco, Monte Mor, Brazil) with controlled lighting (12-h light-dark cycle), humidity (60–80%) and temperature (22 ± 1°C). Water and commercial autoclavable diet (Nuvital, Nuvilab CR1, Curitiba, Brazil) were sterilized by steam and administered *ad libitum*. All experimental procedures were carried out according to the National Council for the Control of Animal Experimentation (CONCEA, 2016). The Ethics Committee in Animal Experimentation (CEUA) from UFMG approved the study (Protocol numbers 96/2011 and 257/2016).

### Bacteria

*Lactobacillus diolivorans* 1Z, previously isolated from watery kefir grains was identified and submitted to previous characterization for probiotic use by *in vitro* assays as described by Zanirati et al. (2015). The strain was lyophilized in 10% (w/v) skim milk (Difco, Sparks, United States) and kept under refrigeration until use. *S. enterica* subsp. *enterica* serovar Typhimurium ATCC 14028 was maintained at −80°C in brain heart infusion broth (BHI, Difco) supplemented with 20% glycerol.

### Experimental Design

In a first set of experiments, CV mice were divided in the two following groups (10 animals each): (CV-CS) treated with water and then challenged with *S.* Typhimurium; and (CV-PS) treated with *L. diolivorans* 1Z and challenged with *S.* Typhimurium. Determination of body weight evolution and survival rate was carried out until 28 days after the pathogenic challenge.

In a second set of experiments, GF mice were divided in the following four groups (16 animals each): (GF) not monoassociated with *L. diolivorans* 1Z and not challenged with *S.* Typhimurium; (GN-CP) monoassociated with *L. diolivorans* 1Z and not challenged with *S.* Typhimurium; (GN-CS) not monoassociated with *L. diolivorans* 1Z and challenged with *S.* Typhimurium; and (GN-PS) monoassociated with *L. diolivorans* 1Z and challenged with *S.* Typhimurium. Four animals of each group were sacrificed after 2, 4, 6, and 8 days of infection. Spleen, liver, feces, small intestinal content, and portions of ileum were collected and used for histopathological and morphometrical analysis and to determinate the ileal and hepatic expression of pro- and anti-inflammatory cytokines, bacterial translocation, fecal counts of bacteria and intestinal sIgA levels.

### *Lactobacillus diolivorans* 1Z Treatment

For CV mice of CV-PS group, lyophilized *L. diolivorans* 1Z cells were ressuspended daily in the drinking water to obtain a concentration of 10^7^ colony forming units (cfu)/ml and offered *ad libitum* during 10 days to mice before the pathogenic challenge. This treatment continued until the end of the experiment. GF mice of GN-CP and GN-PS group received a single intragastric dose of 10^8^ cfu of lyophilized *L. diolivorans* 1Z cells and the monoassociation was maintained during 7 days before the challenge with *S.* Typhimurium for GN-PS mice. CV and GF animals in the CS and GF groups received sterile water during the experimentation period as treatment.

### *Salmonella* Typhimurium Infection

Prior to the challenge, the pathogenic bacterium was grown in BHI broth during 18 h at 37°C. Monoassociated and CV mice were infected intragastrically with 10^2^ and 10^5^ cfu *S.* Typhimurium, respectively (Silva et al., 2004).

### Bacterial Counts in Gnotobiotic Mice

The colonization capacity of *L. diolivorans* 1Z was evaluated by fecal count onto de Man, Rogosa and Sharp agar (MRS, Acumedia, Lasing, United States) as described by Steinberg et al. (2014). To evaluate *in vivo* antagonism, 2, 4, 6, and 8 days after infection, mouse feces were collected by anal stimulation, weighted and suspended in sterile phosphate buffered saline (PBS) to obtain a first 10-fold dilution. Then, serial decimal dilutions were prepared and 0.1 ml of each of them was spread onto MacConkey agar (Acumedia) and incubated at 37°C for 48 h before counts of *Salmonella*. All determinations were made in triplicate and results were expressed as log_10_ cfu/g of feces.

The *ex vivo* antagonistic effect was carried out 4 and 7 days after monoassociation with *L. diolivorans* 1Z by the technique of diffusion in agar as described by Vasconcelos et al. (2003). Briefly, feces of monoassociated mice were collected and layered in a plate containing MRS agar (Acumedia) just before solidification, and then incubated under refrigeration at 4°C for 24 h. After this period, the plate was exposed to chloroform vapor during 30 min when the plates were opened for evaporation of residual chloroform. Then, the plate was overlaid with 3.5 ml of BHI soft agar (0.75%) inoculated with 10^6^ cfu of *S.* Typhimurium and incubated at 37°C for 18 h. The *ex vivo* antagonism was detected by the presence of a growth inhibition zone around the feces.

Bacterial translocation was determined 2, 4, 6, and 8 days after the pathogenic challenge when mice from PS and CS groups were sacrificed. Then, spleen and liver were collected under aseptic conditions, weighted, suspended in sterile PBS to obtain a first 10-fold dilution. Then, serial decimal dilutions were prepared and 0.1 ml of each of them was spread onto MacConkey agar (Acumedia) and MRS agar (Acumedia) and incubated at 37°C during 48 h before counts of *S.* Typhimurium and *L. diolivorans* 1Z, respectively. All determinations were made in triplicate, and results were expressed as log_10_ cfu/g of organ.

### Histopathological and Morphometrical Analysis

For histopathological analysis, 8 days after pathogenic challenge, samples of ileum and liver were collected and fixed in buffered formaldehyde 10% before processing for inclusion in paraffin. The material was processed according to Arantes and Nogueira (1997). From each sample, at least two histological sections (4–5 μm) were stained with hematoxylin and eosin (HE), coded, and analyzed by optical microscopy (Olympus BX51 optical microscope equipped with the Image-Pro Express 4.0 software, Media Cybernetics, United States) by a single pathologist who was unaware of the experimental conditions of each group.

For morphometrical examination, images were made with resolution of 1392 × 1040 pixels and transferred via video camera color Cool SNAP-Proof (Media Cybernetics, Bethesda, MD, United States) to a video system coupled to a computer. To evaluate the ileum, regions of intense inflammatory infiltration in the muscular, with invasion of crypts and destruction of the villi were classified as major lesions. Regions with few inflammatory infiltrations, submucous edema and few crypt destructions were classified as minor lesions. The area perimeter for major and minor lesions was measured by the ImageJ software^1^ (version 1.47 F, Wayne Rasband/National Institutes of Health, United States). Data were normalized and expressed as percentage of regions with each type of lesions per total area. For the liver, quantitative analysis of cellularity using the KS300 software (Zeiss, Jena, Germany). The results were expressed as number of cellular nuclei per mm^2^. Küpffer cells were also counted per 100 hepatocytes.

### Intestinal Secretory Immunoglobulin Type A

The determination of sIgA in the intestinal content was performed by capture ELISA method. Briefly, small intestine of animals was removed and its content was scraped off and collected in 15 ml tubes previously weighed. After collection, the tube was weighted and a volume of PBS supplemented with protease inhibitors (Sigma, St. Louis, MO, United States) (1 μM aprotinin; 25 μM leupeptin, 1 μM pepstatin, and 1 mM of PMSF) was added in a proportion of 2.0 ml to each 500 mg of collected intestinal contents. The mixture was subjected to a cycle of agitation by vortexing, and then centrifuged at 2,000×*g* for 30 min at 4°C. After centrifugation, the supernatant was collected (1.0 ml) and kept at −80°C until analysis. For ELISA procedure, the plate was covered with 100 μl of mouse anti-IgA (Sigma) and incubated overnight at 4°C. Then, 200 μl of blocking solution (1% albumin in PBS Tween) were added and incubated for 1 h at room temperature. The plate was then emptied and washed five times with PBS Tween. Then, 100 μl of the diluted sample (1:1000) were added and the plate incubated for 1 h at room temperature. After incubation, the plate was washed five times with PBS Tween, and 100 μl of conjugate (Sigma) diluted in PBS Tween (10 μg of conjugate in 10 ml of PBS Tween) were added and the plate incubated for an additional 1 h period at room temperature. The plate was emptied and 100 μl of peroxidase substrate (Sigma) added, followed by 40 μl of H_2_O_2_ and incubated for 10 min at room temperature. The reaction was stopped with dilute H_2_SO_4_ (1:20 in distilled water). Reading was performed at 492 nm.

### Cytokine mRNA Expression

Two, four, six, and eight days after infection with *S.* Typhimurium, mice from of all the groups (GF, GN-CP, GN-CS, and GN-PS) were sacrificed for relative expression of mRNA from genes for IL-10, TGF-β, IFN-γ, and TNF-α in ileum and liver, as well as for IL-1β and iNOS only in liver as described by Acurcio et al. (2017), being used as a calibrator of the experiment the GF group. The fragments of ileum and liver measuring 1–2 cm were immersed in RNAlater (Ambion, Austin, TX, United States) and kept at −20°C until total RNA extraction, which was conducted using Trizol (Life Technologies Corp., Grand Island, NY, United States), following the manufacturer’s recommendations. The isolated RNA was submitted to 1% (w/v) agarose gel electrophoresis for integrity evaluation and subsequently quantified in Nanodrop (Thermo Scientific, Inc., Bremen, Germany). Only total RNA samples with more than 200 mg/ml and an A_260_/A_280_ ratio between 1.7 and 2.1 were used. The genomic DNA was removed before the reverse transcription, which was performed with the High capacity cDNA Reverse transcription kit, according to manufacturer’s instructions (Life Technologies, Carlsbad, CA, United States). The resulting cDNA was amplified by RT-qPCR using SYBR Green PCR Master Mix, following the manufacturer’s protocol (Applied Biosystems, Foster City, CA, United States). Gene-specific initiators for cytokines and housekeeping genes for β-actin and glyceraldehyde-3-phosphate dehydrogenase (GAPDH) used as normalizers for the expression data were described by Acurcio et al. (2017). The levels of expression of GF animals were used as calibrators and the relative expression of mRNA for each cytokine was obtained by the derived relative quantification method (Hellemans et al., 2007). The results were expressed as the average and standard deviation of the relative mRNA expression for each cytokine normalized by the expression level of the reference gene.

### Statistical Analysis

The results were analyzed using GraphPad Prism Version 5 (GraphPad software Inc., San Diego, CA, United States), being considered as statistical difference the values with significance level less than 5%. The parametric variables were subjected to the analysis of variance (ANOVA) followed by the Tukey test, for comparison of the averages, and the mortality curves were analyzed by the log-rank survival test.

## Results

**FIGURE 1:**
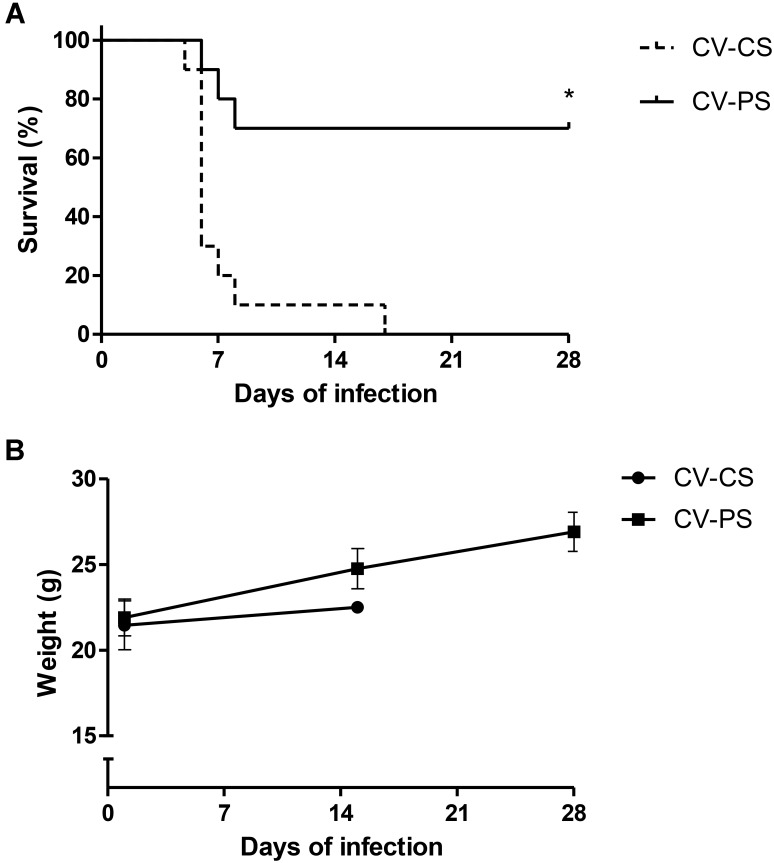
Survival rate **(A)** and body weight evolution **(B)** after oral challenge with *S.* Typhimurium of conventional mice treated (CV-PS) or not (CV-CS) with lyophilized *L. diolivorans* 1Z cells. The symbol “^∗^” indicates statistical difference between groups (Log-rank survival test; *P* < 0.05).

Oral administration of lyophilized *L. diolivorans* 1Z cells to the mice increased the survival rate from 0% for the CV-CS group to 70% for the CV-PS group after 28 days of experiment (Figure 1A) and increased body weight gain of the infected animals (Figure 1B).

**FIGURE 2:**
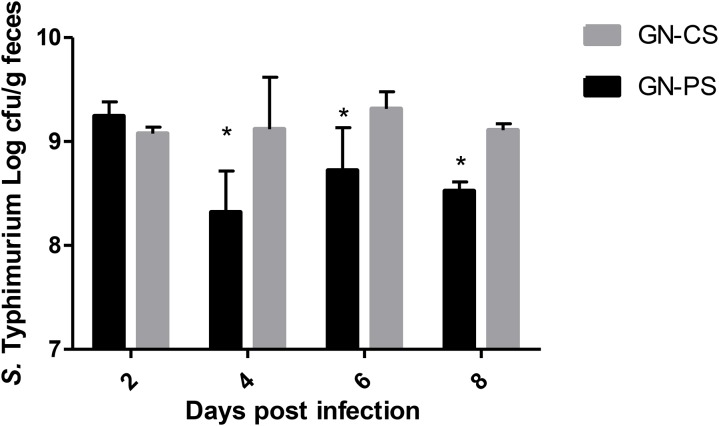
*Salmonella* Typhimurium fecal population levels in gnotobiotic mice monoassociated with *L. diolivorans* 1Z (GN-PS) or not (GN-CS) 2, 4, 6, and 8 days after oral challenge with the pathogen. The symbol “^∗^” indicates statistical difference between groups at each time of infection (ANOVA followed by Tukey test; *P* < 0.05).

After oral inoculation in GF mice, *L. diolivorans* 1Z reached high levels of viable cells in the feces of the monoassociated mice, and the average counts after 4 and 7 days were of 9.52 ± 0.03 and 9.59 ± 0.07 log_10_ cfu/g, respectively (data not shown). Figure 2 shows that, when monoassociated with GF mice (GN-CS group), the population levels of *S.* Typhimurium in the feces reached similar values of about 9.0 log_10_ cfu/g. On the other hand, in diassociated animals (GN-PS group), these levels were significantly reduced (*P* < 0.05). However, *ex vivo* antagonism was not detected around the feces of mice from the PS group before challenge.

**FIGURE 3:**
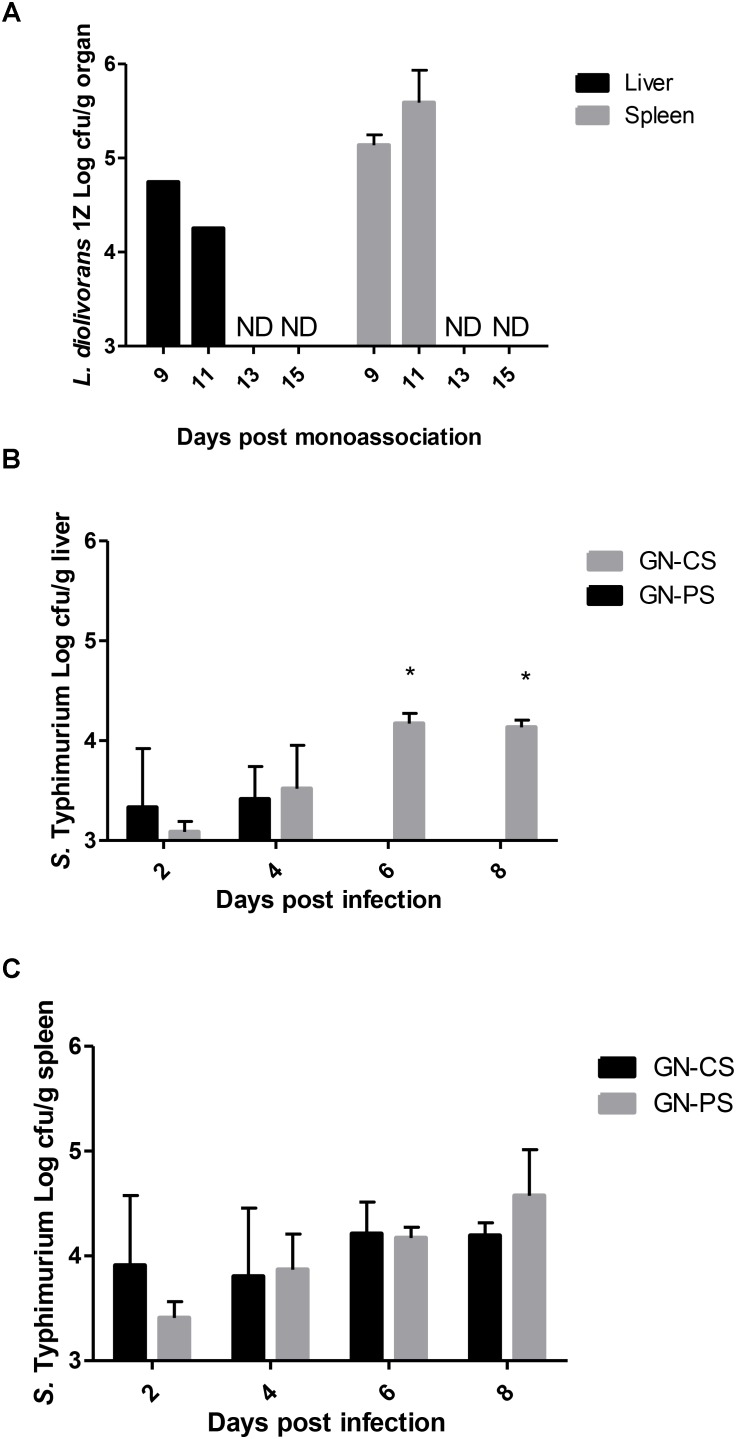
Translocation of *L. diolivorans* 1Z to liver and spleen **(A)** and of *S.* Typhimurium in liver **(B)** and spleen **(C)** of gnotobiotic mice monoassociated with *L. diolivorans* 1Z (GN-PS) or not (GN-CS), after 2, 4, 6, and 8 days of infection. The symbol “^∗^” indicates statistical difference between groups at each time of infection (ANOVA followed by Tukey test; *P* < 0.05). ND, not detected.

Figure 3A shows that after its monoassociation, *L. diolivorans* 1Z translocated from the intestines to liver and spleen, reaching values of 4.25 ± 0.93 and 5.58 ± 0.35 log_10_ cfu/g of organ, respectively, on the 11th day of monoassociation (GN-CP group). However, by day 13, the population levels of *L. diolivorans* 1Z in these organs fell to values undetectable by the method (<3.00 log_10_ cfu/g). Concerning *S.* Typhimurium population levels in the liver, a gradual increase was observed in the GN-CS group during the time of infection, whereas low levels were only detected until day 4 of challenge in GN-PS (Figure 3B). In the spleen, similar population levels were observed in both groups during all the experiment (Figure 3C).

**FIGURE 4:**
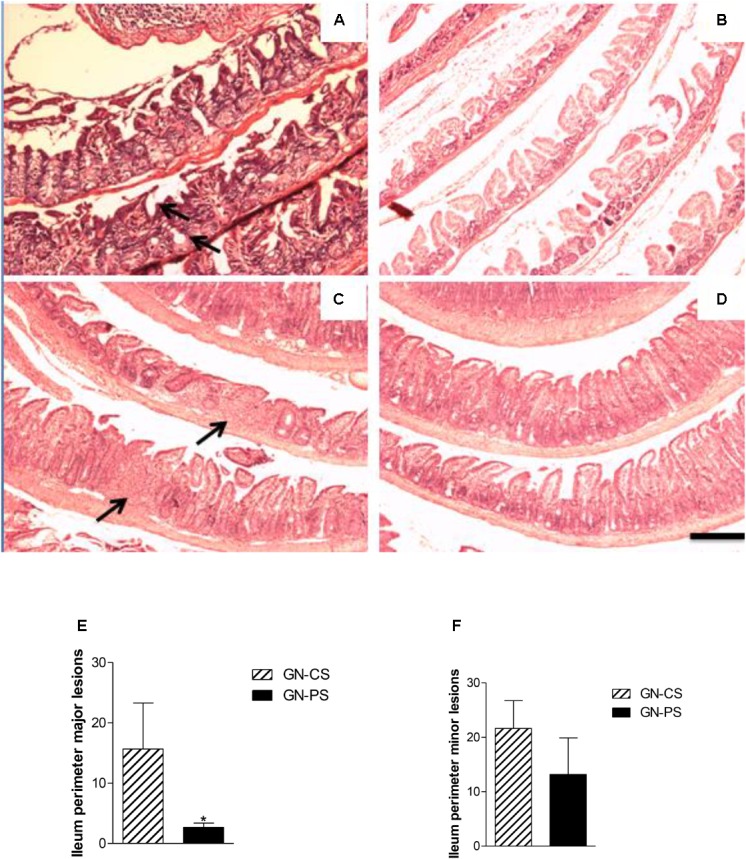
Histopathological aspect of ileum from mice monoassociated with *L. diolivorans* 1Z (GN-PS) or not (GN-CS) and challenged with *S.* Typhimurium. **(A)** GN-CS 2d, and **(B)** GN-PS 2d, animals sacrificed after 2 days of infection; **(C)** GN-CS 8d and **(D)** GN-PS 8d, animals sacrificed after 8 days of infection. Arrows indicate areas of villus damage with discreet increase of the cellularity of the lamina propria in panel **(A)** and inflammatory infiltrate focus in panel **(C)**. H&E, scale bar represents 100 μm. Perimeter of minor **(E)** and major **(F)** lesions attributed to *Salmonella* infection in ileum of mice monoassociated (GN-PS) or not (GN-CS) with *L. diolivorans* 1Z and challenged with *S.* Typhimurium. The samples were taken at day 8 postinfection. The symbol “^∗^” indicates statistical difference between the groups GN-CS and GN-PS (ANOVA followed by Tukey test; *P* < 0.05).

Changes in the ileum of GN-CS mice after 2 days of infection with *S.* Typhimurium were predominantly represented by discrete edema of the lamina propria, epithelial vacuolization, flaking of the villus top and presence of some intraepithelial lymphocytes. These lesions progressed with polymorphonuclear inflammatory infiltrates and epithelial necrosis, accompanied by lamina propria enlargement at the villus top (Figure 4A). On day 8 of infection, more discreet lesions were noticed, with traces of epithelial regeneration (Figure 4C). Two days after infection, some mice of the GN-PS group presented very discreet alterations of the ileum mucosa, with few superficial infiltrates and edema areas and other animals did not showed any noteworthy changes (Figure 4B). By day 8, a preservation of the epithelium was observed, despite small areas with increased cellularity (Figure 4D). No differences were observed in the perimeter of minor lesions in the ileum between the animals of the groups GN-CS and GN-PS at day 8 after challenge (*P* > 0.05) (Figure 4E). On the other hand, major lesions in the ileum were lower in the animals of the GN-PS group when compared to the group GN-CS at the same time (*P* < 0.05) (Figure 4F).

**FIGURE 5:**
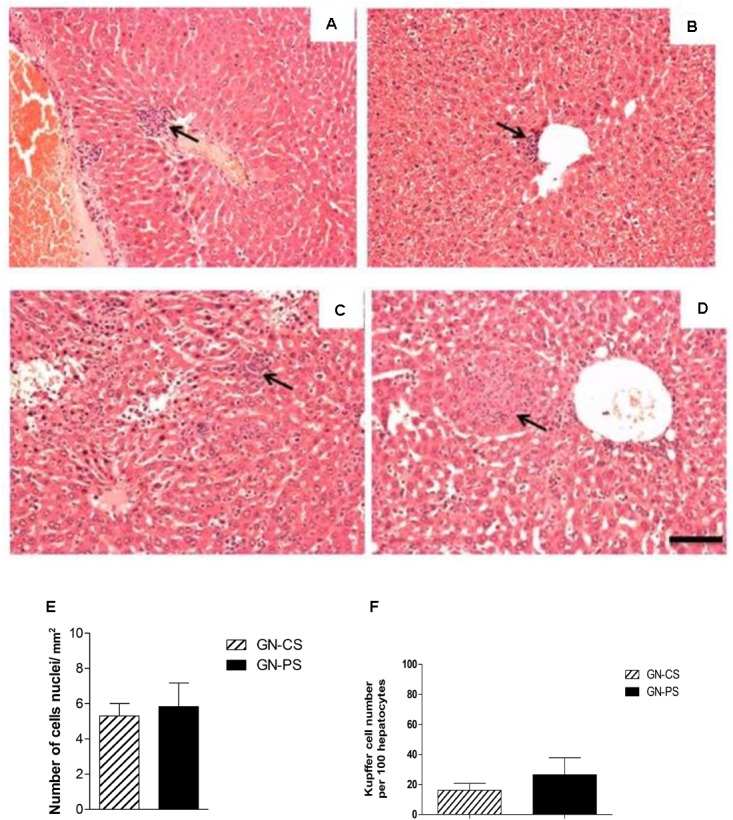
Histopathological aspect of liver from mice monoassociated with *L. diolivorans* 1Z (GN-PS) or not (GN-CS) and challenged with *S.* Typhimurium. **(A)** GN-CS 2d, and **(B)** GN-PS 2d, animals sacrificed after 2 days of infection; **(C)** GN-CS 8d and **(D)** GN-PS 8d, animals sacrificed after 8 days of infection. Arrows indicate areas of inflammatory infiltrate. H&E, scale bar represents 100 μm. **(E)** Liver cellularity (cells nuclei/mm^2^), and **(F)** number of Küpffer cells per 100 hepatocytes in the animal liver of mice monoassociated (GN-PS) or not (GN-CS) with *L. diolivorans* 1Z and challenged with *S.* Typhimurium. The samples were taken at day 8 postinfection. The symbol “^∗^” indicates statistical difference between the groups GN-CS and GN-PS (ANOVA followed by Tukey test; *P* < 0.05).

The livers of GN-CS group also presented progressive changes over time of infection, beginning on day 2 of infection, with mononuclear and polymorphonuclear cell infiltration restricted to small foci and with relative preservation of the parenchyma (Figure 5A). These findings became more intense and diffuse in the liver of animals sacrificed by day 8, with large areas of necrosis accompanied by multiple foci of inflammatory polymorphonuclear infiltration (Figure 5C). Two days after the infection, livers from animals of the PS group presented an aspect very similar to GN-CS group (Figure 5B). Rare foci of predominantly mononuclear infiltration were observed and some of the animals did not present any changes. On day 8, liver of the animals of the GN-PS group presented predominantly polymorphonuclear infiltrates smaller than in the animals of the GN-CS group with discreet degenerative changes of the parenchyma (Figure 5D) and a well preserved hepatic parenchyma. There was no significant difference in cellularity in liver between animals of the GN-PS and GN-CS groups (*P* > 0.05) (Figure 5E).

**FIGURE 6:**
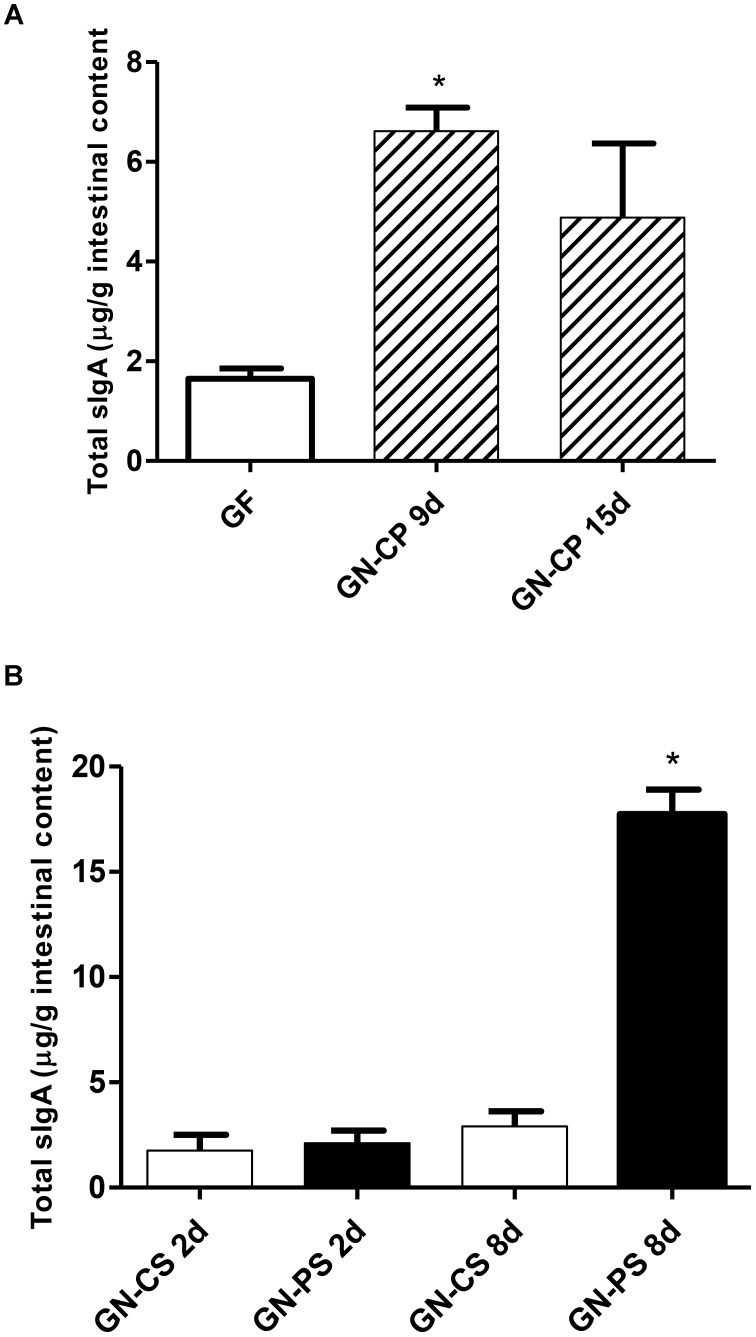
**(A)** Secretory immunoglobulin A levels in the intestinal fluid of germ-free (GF) and gnotobiotic mice monoassociated with *L. diolivorans* 1Z (GN-CP) after 9 and 15 days of monoassociation. The symbol “^∗^” indicates statistical difference between groups (*P* < 0.05). **(B)** Secretory immunoglobulin A levels in the intestinal fluid of gnotobiotic mice monoassociated with *L. diolivorans* 1Z (GN-PS) or not (GN-CS) after 2 and 8 days of infection. The symbol “^∗^” indicates statistical difference between groups (ANOVA followed by Tukey test; *P* < 0.05).

The monoassociation with *L. diolivorans* 1Z induced production of sIgA in the intestinal fluid when compared to GF mice, although statistically difference was found only 9 days after monoassociation (Figure 6A). Interestingly, sIgA concentration in the intestinal fluid of mice from GN-PS group was higher when compared to GN-CS group and significantly different by day 8 after infection (Figure 6B).

**FIGURE 7:**
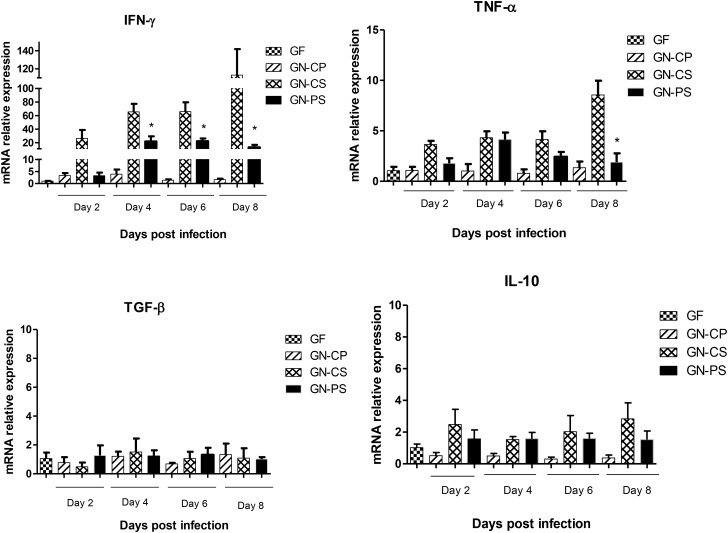
Relative levels of mRNA of genes encoding cytokines, IFN-γ, TNF-α, TGF-β, and IL-10 in ileum of mice monoassociated or not with *L. diolivorans* 1Z and challenged or not with *S.* Typhimurium. GF, germ-free mice without challenge; GN-CP, germ-free mice monoassociated with *L. diolivorans* 1Z without challenge; GN-CS, germ-free mice challenged with *S.* Typhimurium; GN-PS, germ-free mice monoassociated with *L. diolivorans* 1Z and challenged with *S.* Typhimurium. The symbol “^∗^” indicates statistical difference between groups (ANOVA followed by Tukey test, *P* < 0.05).

In the ileum, there was a progressive increase in the expression of IFN-γ and TNF-α genes in GN-CS group from days 2 to 8 of infection. This increase was reduced in GN-PS group, with statistical difference (*P* < 0.05) from the second day of infection for IFN-γ and only on the eighth day for TNF-α. For TGF-β and IL-10 there was no statistical difference between the groups during the infection period (Figure 7).

**FIGURE 8:**
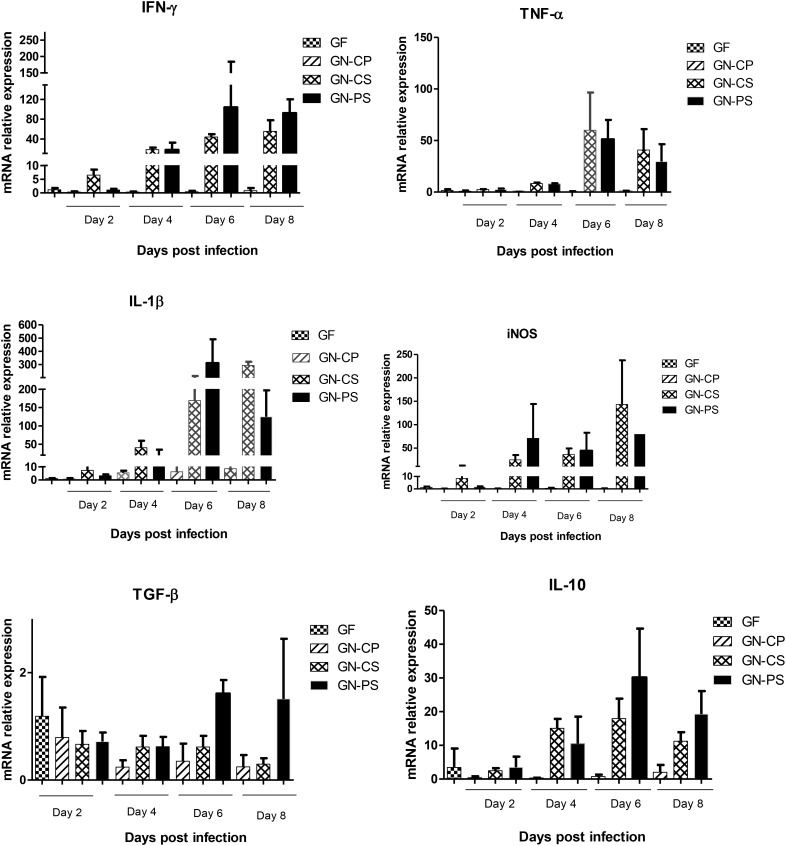
Relative levels of mRNA of genes encoding cytokines IFN-γ, TNF-α, IL1-β, TGF-β, and IL-10 and the enzyme iNOS in liver of mice monoassociated or not with *L. diolivorans* 1Z and challenged or not with *S.* Typhimurium. GF, germ-free mice without infection; GN-CP, germ-free mice monoassociated with *L. diolivorans* 1Z without infection; GN-CS, germ-free mice challenged with *S.* Typhimurium; GN-PS, germ-free mice monoassociated with *L. diolivorans* 1Z and challenged with *S.* Typhimurium. The symbol “^∗^” indicates statistical difference between groups (ANOVA followed by Tukey test; *P* < 0.05).

In the liver, there was a progressive increase in the expression of the cytokines IFN-γ, TNF-α and IL-1β, TGF-β, IL-10, and enzyme iNOS in the GN-PS and GN-CS groups until day 6, followed by a tendency to a decrease in the expression of hepatic TNF-α, IL1-β, and iNOS in PS group when compared with the GN-CS group. A simultaneous, but not statistically different, increase in the expression of the regulatory cytokines TGF-β and IL-10 was observed in the GN-PS group by days 6 and 8 (Figure 8).

## Discussion

In the present study, higher survival (70%) and body weight gain were observed in animals that were previously treated with oral administration of *L. diolivorans* 1Z in comparison with animals receiving only water (0% survival) before the pathogenic challenge. Probiotics can exert their beneficial effects by means of two main mechanisms of action which are colonization resistance and host immunomodulation.

In the case of colonization resistance, probiotics can act by competing for nutrients and adhesion receptors or producing inhibitory compounds, such as organic acids and bacteriocins (Lebeer et al., 2008). Using a murine gnotobiotic model, a reduction in the number of *Salmonella* in feces of the GN-PS group was observed when compared to the GN-CS group. *L. diolivorans* 1Z inhibited the growth of *S.* Typhimurium *in vitro* (Zanirati et al., 2015), but the same effect was not detected in the *ex vivo* assay tested in the present study. Three hypothesis could explained these results: (1) the reduction of *Salmonella* populations could be due to nutrient competition; (2) the production of an inhibitory compounds was induced *in vitro* (MRS medium) but not *ex vivo* (intestinal contents); and (3) the inhibitory compounds was produced in the small intestine and degraded in the large intestine. The gnotobiotic model showed another important characteristic for potential probiotic according to FAO/WHO (2002): the capacity to pass in a viable form through the stressful conditions of the gastrointestinal environment without pathogenic consequences for the host. *L. diolivorans* 1Z was able to colonize and to maintain population levels of about 10^9^ cfu/g of feces during the experiment (data not shown). Additionally, the safety of *L. diolivorans* 1Z was demonstrated by data of translocation to liver and spleen (Figure 3A), histopathological analysis of liver and ileum (data not shown), and mRNA expression of genes for anti- and pro-inflammatory cytokines in the mice monoassociated with *L. diolivorans* 1Z (Figures 6, 7). Translocation of a microorganism is defined as the passage of viable cells through the mucous barrier to extraintestinal sites, such as mesenteric lymph nodes, liver and spleen. Since 1995, Rodney Berg proposed that a physiological translocation might be responsible for host immunomodulation by indigenous microbiota (Berg, 1995). Similarly, controlled translocation of a probiotic could activate antigen presentation events and local or systemic immune modulation and could be beneficial to the host. On the other hand, uncontrolled translocation of pathogenic microorganisms, such as *Salmonella*, leads to sepsis and death. A progressive decrease in the hepatic levels of *S.* Typhimurium was observed in the GN-CS group, whereas *S.* Typhimurium was detected in low counts at the beginning of the infection and then disappeared in the GN-PS group. This finding, allied to the initial transient translocation of *L. diolivorans* 1Z to the liver of the monoassociated animals, suggest that the bacterium could increase the number of Küpffer cells, such as described by Neumann et al. (1998) with *L. acidophilus* UFV-H2b20 and Martins et al. (2011) with *Saccharomyces cerevisiae* UFMG 905, which associated the increase of Küpffer cells with the elimination of *Escherichia coli* B41 and *S.* Typhimurium, respectively. In fact, a histological analysis of the liver revealed an increase, but not statistically different, in the Küpffer cells in the GN-PS group (Figure 5F).

The histopathological analysis of the ileum of mice from the GN-CS group showed changes of the ileum mucosa that progressed and were intensifying from the second day postinfection. In the GN-PS group, these changes were initially also observed, but with less intensity or absent in some animals, and the protection in the GN-PS group, compared to the GN-CS group, was confirmed by the significant lower perimeter of serious lesions due to the infection.

Just like in the intestine, the hepatic lesions increased during the infection, and in both GN-CS and GN-PS groups, areas of inflammatory infiltration were observed with degenerative changes of the hepatic parenchyma. The administration of the *L. diolivorans* 1Z did not prevent the initial low translocation of *S.* Typhimurium to the liver, which can explain some hepatic lesions observed in the GN-PS group. However, later, the pathogen counts gradually decreased, which can explain the earliest recovery of hepatic lesions in the GN-PS group when compared to the GN-CS group. The presence of the pathogen in the liver suggests that there may have been an increase in the recruitment of neutrophils and monocytes and the maintenance of an inflammatory response in the tissue. Apparently the hepatic inflammatory response was sufficient to eliminate the pathogen, avoiding sepsis and death of the animal in the GN-PS group. Although cellularity was similar in both groups, it is important to say that the method of evaluation of this parameter did not discriminate against the type of leukocyte present in the inflammatory infiltrate. However, a qualitative analysis realized in the liver showed that in the GN-CS group the most prevalent type of inflammatory cells in the infiltrate was of polymorphonuclear, while in the GN-PS group prevailed mononuclear cells, indicating that the inflammation in the liver of the animals between the groups was in different phases. It is already well described in the literature that the first cells to migrate to the infection site are the neutrophils (polymorphonuclear) that phagocytose the pathogen and then die by apoptosis. Subsequently, the monocytes are recruited to phagocyte the apoptotic cells remaining in the tissue, which results in the resolution of inflammation (Abbas et al., 2015). Thus, it can be hypothesized that the administration of *L. diolivorans* 1Z contributed to a more rapid inflammatory response in the liver and, therefore, a more efficient elimination of *S.* Typhimurium.

Local and systemic immune mechanisms could explain the higher survival and the reduced translocation and histopathological lesions observed in animals of the GN-PS group in relation to the GN-CS group. In this regard, some authors have demonstrated that probiotic strains might increase the production of sIgA and phagocytic activity of macrophages, reducing infections (Leblanc et al., 2010; Maciel et al., 2016).

Immunoglobulin A is the predominant antibody in the intestinal lumen where plays a crucial role in preventing infection by trapping pathogenic bacteria in the mucus layer (Fransen et al., 2015). Moreover, the sIgA binding can alter the expression of flagelin which compromises motility and reduces the virulence of some microorganisms such as *Salmonella* (Cullender et al., 2013). Finally, the sIgA neutralizes the action of bacterial toxins, protecting the epithelial cells of the toxic damage (Mantis et al., 2011; Gutzeit et al., 2014). The sIgA concentration in the intestinal fluid of the animals of the GN-PS and GN-CS group was statistically equal in the first day after the infection, but higher in the GN-PS group on the eighth day after the challenge. It could be suggested that the sIgA increase in the intestinal would contained the translocation of the pathogen and the increase of Küpffer cells could eliminated salmonella that had already translocated. Similar results have been already found with others potential probiotics (Alvim et al., 2016; Acurcio et al., 2017).

In intestinal infections such as salmonellosis, there is an increase in IL-6 and IFN-γ levels, which stimulates an inflamed environment favorable to *Salmonella* multiplication and pathogenicity but impairs components of the indigenous microbiota (Kuzminska-Bajor et al., 2015). Indeed, the promotion of reactive oxygen species (ROS) liberation by inflamed epithelial cells will deplete commensal microbiota whereas will harm only discretely the *Salmonella* population (Thiennimitr et al., 2012). This is given mainly due to its use, as respiratory electron acceptor, of sulfur compounds (such as tetrathionate) formed by ROS action, that is not possible by anaerobic indigenous bacteria (Winter et al., 2010). During the infection of mice with *S.* Typhimurium, the first cells found by the pathogen are the intestinal epithelial cells, dendritic cells (DCs) and macrophages (Murphy, 2014). The interaction with these cells induces the synthesis of inflammatory cytokines, such as TNF-α and IFN-γ, leading to a massive influx of immature neutrophils, macrophages and DCs, which are necessary for the suppression of bacterial growth in intestinal lumen in *Salmonella* infections (Mastroeni and Grant, 2011). In the present study, the experimental infection produced an increase in the pro-inflammatory cytokines IFN-γ and TNF-α in the ileum of both GN-CS and GN-PS groups when compared to GF and GN-CP groups. However, this increase was significantly lower in the GN-PS group and was accompanied by a decrease in the count of *S.* Typhimurium in the feces. Since the expression of these cytokines depends on the contact between the pathogen and the cells of the intestinal epithelium, such as DCs and macrophages, it is to be expected that a lower level of pathogenic bacteria in the intestinal lumen resulted in less expression of these cytokines. The action of TNF-α throughout the inflammatory process is paradoxical. Increased levels of this cytokine at the onset of inflammation are important for the containment of a pathogenic microorganism at the site of infection. However, if this protective mechanism fails and high levels of TNF-α were maintained in the intestines, this can compromise the function of epithelial barrier by increasing intestinal permeability, which facilitates the pathogen translocation (Beaurepaire et al., 2009; Murphy, 2014). In GN-CS group, the uninterrupted increase in TNF-α and IFN-expression may have compromised the epithelial barrier and thus facilitated the translocation of the *S.* Typhimurium to liver and spleen, contrarily to the GN-PS group where the decrease in the expression of TNF-α and IFN-γ may be associated with improved preservation of intestinal epithelial integrity and resistance to translocation of the pathogen, which were observed in the administration of *L. diolivorans* 1Z.

The increase in the gene expression of IFN-γ, TNF-α, IL1-β, and iNOS in the liver of both groups after the infection is indicative of a systemic dissemination of *S.* Typhimurium which stimulated the hepatic macrophages to produce these pro-inflammatory cytokines (Murphy, 2014). The systemic release of TNF-α in the bloodstream causes vasodilation and increased vascular permeability, causing loss of plasma volume and, finally, septic shock, leading to a breakdown in the function of several vital organs, such as kidney, liver, and lung, being usually the cause of death of animals infected with *S.* Typhimurium (Murphy, 2014; Abbas et al., 2015). Mice with defects or absence of TNF-α receptors are resistant to septic shock but unable to control infection. This fact illustrates the paradoxical role of TNF-α, important to contain infection in the primary site of infection, but lethal when released systemically in the inflammatory response induced by Gram-negative bacteria (Pfeffer, 2003). The tendency to a decrease in the expression of hepatic TNF-α, IL1-β, and iNOS was observed in GN-PS group when compared with the GN-CS group, and this could be explained by the simultaneous increase in the expression of the regulatory cytokines TGF-β and IL-10 (Murphy, 2014). In a review, Souza and Teixeira (2005) said that the balance between the production of TNF-α and IL-10 determines tissue injury after ischemia and intestinal reperfusion. The authors also highlighted that therapeutic strategies that increase the IL-10 and reduce the concentration in TNF-α could be used as adjuvants for treatment of such tissue injuries, as well as for infectious diseases. In this context, the use of probiotics could be an alternative. Silva et al. (2004) demonstrated that the protection against *S.* Typhimurium infection of animals treated with *Bifidobacterium longum* Bb46 could be attributed in part to the decrease in the production of IFN-γ and by the increase of IL-10. In addition, the increase in IFN-γ in liver of GN-PS group mice may be indicative of the largest microbicidal activity of the macrophages at the site of the infection, since IFN-γ from NK cells increases the capacity of macrophages to kill phagocyted bacteria (Sun and Lanier, 2011). This interaction between INFγ and macrophage-dependent NK cells may be sufficient to control infection by intracellular bacteria such as *Listeria monocytogenes* and *S.* Typhimurium (Abbas et al., 2015).

Concluding, the previous oral administration of *L. diolivorans* 1Z was able to protect animals against an oral infection with *S.* Typhimurium, as demonstrated by the higher survival in conventional mice, and the lower translocation and histopathological lesions in GN mice. Colonization resistance as well as local and systemic immunomodulations seem to be the mechanisms involved in these protective effects. The potential of *L. diolivorans* 1Z as a probiotic is enhanced by its successfully and safety use in a lyophilized form.

## Author Contributions

EN and AN designed the study. MJ conducted all the experiments. SS helped with the cytokine mRNA detection. MR and RA were responsible for the histological analysis. MJ, EN, JN, and AN interpreted and critically revised the data. EN and JN prepared the manuscript to submission with the help of all authors.

## Conflict of Interest Statement

The authors declare that the research was conducted in the absence of any commercial or financial relationships that could be construed as a potential conflict of interest.

## Footnotes

1 http://rsbweb.nih.gov/ij/download.html
